# Effects of Language, Age, and Hearing Loss on Health‐Related Quality of Life

**DOI:** 10.1002/oto2.55

**Published:** 2023-05-28

**Authors:** Harrison J. Ma, Francis Reyes Orozco, Christine K. Raj, Kevin Herrera, John C. Parsons, Ian Kim, Kevin Hur

**Affiliations:** ^1^ Caruso Department of Otolaryngology–Head and Neck Surgery, Keck School of Medicine University of Southern California Los Angeles California USA; ^2^ Department of Population and Public Health Sciences, Keck School of Medicine University of Southern California Los Angeles California USA

**Keywords:** aging, health outcomes, health‐related quality of life, hearing loss, language, otology

## Abstract

**Objective:**

To understand the effect of age on health‐related quality of life (HRQoL) in patients with hearing loss and determine how primary language mediates this relationship.

**Study Design:**

Cross‐sectional study.

**Setting:**

General otolaryngology clinic in Los Angeles.

**Methods:**

Demographics, medical records, and HRQoL data of adult patients presenting with otology symptoms were reviewed. HRQoL was measured using the Short‐Form 6‐Dimension utility index. All patients underwent audiological testing. A path analysis was performed to generate a moderated path analysis with HRQoL as the primary outcome.

**Results:**

This study included 255 patients (mean age = 54 years; 55% female; 27.8% did not speak English as a primary language). Age had a positive direct association with HRQoL (*p* < .001). However, the direction of this association was reversed by hearing loss. Older patients exhibited significantly worse hearing (*p* < .001), which was negatively associated with HRQoL (*p* < .05). Primary language moderated the relationship between age and hearing loss. Specifically, patients who did not speak English as a primary language had significantly worse hearing (*p* < .001) and therefore worse HRQoL (*p* < .01) than patients who spoke English as a primary language with hearing loss. Increasing age was associated with bilateral hearing loss compared to unilateral hearing loss (*p* < .001) and subsequently lower HRQoL (*p* < .001). Polypharmacy (*p* < .01) and female gender (*p* < .01) were significantly associated with lower HRQoL.

**Conclusion:**

Among otolaryngology patients with otology symptoms, older age and not speaking English as a primary language were associated with worse hearing and subsequently lower HRQoL.

Age is one of the strongest predictors of hearing loss.^1^, ^2^, ^3^ The prevalence of hearing loss increases from 2% to 50% as adults age from 45 to over 75 years.^4^ Studies have also shown that hearing loss has a strong negative influence on health‐related quality of life (HRQoL) in geriatric populations.^5^, ^6^, ^7^, ^8^ In particular, bilateral hearing loss (BHL) was associated with significantly lower HRQoL^7^ with profound negative consequences for the social, functional, and psychological well‐being of a person.^8^ However, no previous studies have investigated how age affects the synchronous effect between hearing loss and HRQoL.

While the extent and impact of aging on hearing loss may differ from person to person,^1^, ^2^, ^3^ patients who do not speak the same language as their physicians have experienced processes and outcomes of care worse than those provided to patients who speak the same language as their physicians.^9^, ^10^, ^11^ These studies suggest that language barriers may result in delayed or lower quality medical care for hearing loss in patients who do not speak English as a primary language, which is in turn associated with lower HRQoL.^12^ Despite the severity of this issue, no published studies have examined the role of a patient's primary language on the relationship between age, hearing loss, and HRQoL.

This study addresses these gaps by investigating the effects of hearing loss on the association between age and HRQoL and examining the role of primary language on hearing loss and HRQoL among patients with otology symptoms. We hypothesized that (1) hearing loss will mediate the association between age and HRQoL, and (2) the negative impact of aging and hearing loss on HRQoL will be amplified in patients who do not speak English as a primary language.

## Methods

### Sample and Procedures

Data was collected from a chart review of patients aged 18 years and older at a general otolaryngology clinic in Los Angeles who underwent audiological testing and reported 1 or more otology symptoms including hearing loss, tinnitus, ear pressure, vertigo, dizziness, otalgia, ear pruritus, otorrhea, and phonophobia. Patients with otology symptoms who did not undergo audiological testing were excluded from the study. The study was approved by the Institutional Review Board at the University of Southern California.

### Sociodemographic and Clinical Information

Demographic covariates collected from medical records included age (years), self‐reported gender (male, female, other), self‐reported ethnicity (Hispanic or Latino, not Hispanic or Latino), self‐reported race (white or Caucasian, African American or black, American Indian or Alaska Native, Native Hawaiian or Pacific Islander, Asian, other), and primary language (English, Spanish, Mandarin, Cantonese, other). To determine one's primary language, the physician asked for each patient's primary language at the start of the encounter and documented the response in the medical chart.^13^ Patients who did not speak English fluently were given the option of using a certified translator in the patient's primary language either in person or via video conferencing during the visit. Some physicians were fluent in Mandarin or Spanish and therefore some patients may not have used a translator during those visits but were still offered the option of using a certified translator. Primary languages were then classified as “English,” “Spanish,” “Mandarin,” “Cantonese,” or “Other.” Patient medical records were further examined for clinical covariates such as home medications and otologic symptoms.

### Hearing Measurement

Speech‐frequency pure‐tone averages (PTAs) were used to measure hearing loss severity. Audiometric testing was performed in English. The audiologist explained the test using a certified translator. The speech‐frequency PTA was calculated across 3 test frequencies: 0.5, 1, and 2 kHz. Separate PTAs were calculated for each ear, with the worse ear used to determine PTA.^14^ Hearing loss severity was determined by various PTA thresholds (≤25 dB = normal hearing; 25‐40 dB = mild hearing loss; 40‐55 dB = moderate hearing loss; 55‐70 dB = moderately severe hearing loss; 70‐90 dB = severe hearing loss; >90 dB = profound hearing loss).^2^ Hearing loss was categorized by laterality: no hearing loss, unilateral hearing loss (UHL), and BHL. UHL was defined according to the World Health Organization World Report on Hearing.^15^ Patients who had a PTA ≥ 35 dB in one ear and PTA ≤ 25 dB in the other ear were considered to have UHL. Patients who had PTA > 25 dB in both ears were considered to have BHL.

### Main Outcome

The main outcome of this study was HRQoL, which was calculated using responses to the 12‐Item Short‐Form Survey (SF‐12), which has been used in previous studies to determine the HRQoL in patients with hearing loss.^16^, ^17^, ^18^ Patients completed the SF‐12 on the day of their visit to measure their HRQoL at the time of their symptoms. The SF‐12 is an abbreviated version of the 36‐Item Short‐Form Survey.^16^ Responses from the SF‐12 were transformed into the Short‐Form 6‐Dimension (SF‐6D) and scored according to the algorithm developed by the Department of Health Economics and Decision Science at the University of Sheffield, via the same method employed by Rudmik et al^19^ SF‐6D scores ranged from 0.0 to 1.0, with 0 indicating death and 1 indicating perfect health. The SF‐12 was distributed in English only. Patients who did not speak English as a primary language were aided by physicians who spoke were able to speak the same language, certified translators, or hospital staff who spoke the same language.

### Statistical Analysis

A priori power analysis was performed using G*Power given the number of variables in the model, the anticipated effect size, and the desired probability to reach the statistical power level of 0.80 (N = 76). Patient characteristics were summarized using descriptive statistics. Once a sample of each categorical variable group was drawn, race was excluded from further analysis because not all groups met the minimum required sample size in each category (n = 5). To evaluate the hypothesized relationships, we performed a path analysis model using the *Lavaan* package in R Studio 1.3.1.^20^ Statistically significant covariate variables were included in modeling to test the impact of PTA, hearing loss laterality, age, and primary language on HRQoL, with an interaction term between age and primary language. A path analysis is a sequential multiple regression analysis, where its equations are solved simultaneously to decompose the total effect of independent variables. These effects are then classified as direct or indirect effects. Direct effects affect outcome variables without an intermediary variable, while indirect effects affect outcome variables through an intermediary variable.^21^ All paths in the model were adjusted for gender, ethnicity, the number of medications, the number of other otologic symptoms, and the duration of the symptoms. Patients with missing data were excluded from the analysis. Indirect effects in this model were based on a bootstrapping analysis of 1000 iterations. The final path model contained only parameters that were statistically significant, thus nonsignificant parameters were removed to yield a parsimonious final model.^22^ Model fit was evaluated using the global *χ* ^2^ test and root mean square error of approximation (RMSEA) statistics in combination with the comparative fit index (CFI) and standardized root mean square residual (SRMR).^23^ The strengths of relationships between study variables were estimated using standardized path coefficients.

## Results

Participants were primarily female (n = 139, 55%), non‐Hispanic or Latino (n = 201, 83%), and ranged in age from 18 to 90 (mean age = 54.0 ± 18.4 years) years. Almost one‐third (27.8%) of the patients did not speak English as their primary language (Spanish 2.3%, Mandarin 19.6%, Cantonese 3.1%, Other 2.8%). Mean HRQoL was similar between patients who did not speak English as a primary language and patients who spoke English as a primary language (0.75 ± 0.13 vs 0.74 ± 0.12). Regarding hearing levels, PTA scores were significantly higher in patients who did not speak English as a primary language than in patients who spoke English as a primary language (38.48 ± 19.71 vs 27.86 ± 20.91; *p* < .001). Almost half (48%) of the patients had hearing loss on the audiogram. A significantly greater proportion of patients who did not speak English as a primary language had moderate to profound hearing loss compared to patients who spoke English as a primary language (23.6% vs 9.3%; *p* < .001). Patients who spoke English as a primary language had a significantly lower proportion of BHL, compared to patients who did not speak English as a primary language (23% vs 54%; *p* < .001). Demographic characteristics from the study sample are presented in Table 1.

**Table 1:** Demographic Characteristics of the Study Sample (N = 255)

	Primary language group			
Characteristic	English (n = 184)^a^	Non‐English (n = 71)^a^	Total (N = 255)^a^	*p* b
Age, years	54.1 (18.3)	68.5 (12.5)	58.1 (18.1)	<.001
Gender				.521
Male	86 (47%)	30 (42%)	116 (45%)	
Female	98 (54%)	41 (58%)	139 (55%)	
Hispanic or Latino	37 (20%)	6 (8.5%)	43 (17%)	.031
Race				<.001
White or Caucasian	62 (34%)	2 (2.8%)	64 (25.1%)	
African American or black	11 (6.0%)	0 (0%)	11 (4.3%)	
American Indian or Alaska Native	1 (0.5%)	0 (0%)	1 (0.4%)	
Native Hawaiian or Pacific Islander	1 (0.5%)	0 (0%)	1 (0.4%)	
Asian	54 (29%)	64 (90%)	118 (46.3%)	
Other	55 (30%)	5 (7.0%)	60 (23.5%)	
Number of medications	4.33 (4.32)	4.89 (4.15)	4.49 (4.27)	.353
Number of symptoms	1.95 (0.95)	2.01 (0.85)	1.97 (0.92)	.595
Hearing loss (HL)	124 (67%)	55 (77%)	179 (70%)	
Tinnitus	84 (46%)	36 (51%)	120 (47%)	
Ear pressure aural fullness	60 (33%)	14 (19%)	76 (30%)	
Vertigo	17 (9.2%)	8 (11%)	25 (9.8%)	
Dizziness	14 (7.6%)	4 (5.6%)	18 (7.1%)	
Otalgia	40 (22%)	11 (15%)	51 (20%)	
Ear pruritus	3 (1.6%)	4 (5.6%)	7 (2.7%)	
Otorrhea	4 (2.2%)	5 (7.0%)	9 (3.5%)	
Phonophobia	5 (2.7%)	0 (0%)	5 (2.0%)	
Duration of symptoms				.110
<1 wk	21 (11%)	6 (8.5%)	27 (11%)	
1 wk to 1 mo	42 (23%)	10 (14%)	52 (20%)	
1‐6 mo	51 (28%)	14 (20%)	65 (25%)	
6 mo to 1 y	13 (7.1%)	8 (11%)	21 (8.2%)	
1‐3 y	19 (10%)	14 (20%)	33 (13%)	
>3 y	38 (21%)	19 (27%)	57 (22%)	
PTA	27.90 (20.86)	38.48 (19.71)	30.84 (21.05)	<.001
Severity				<.001
No HL	109 (60%)	23 (32%)	132 (52%)	
Mild: 25‐40 dB HL	36 (20%)	15 (21%)	51 (20%)	
Moderate: 40‐55 dB HL	22 (12%)	16 (23%)	38 (15%)	
Moderate‐severe: 55‐70 dB HL	7 (3.8%)	13 (18%)	20 (7.8%)	
Severe: 70‐90 dB HL	6 (3.3%)	4 (5.6%)	10 (3.9%)	
Profound: >90 dB HL	4 (2.2%)	0 (0%)	4 (1.6%)	
Hearing loss laterality				<.001
None	124 (67%)	28 (39%)	152 (60%)	
Unilateral	12 (6.5%)	5 (7.0%)	17 (6.7%)	
Bilateral	48 (26%)	38 (54%)	86 (34%)	

Abbreviation: PTA, pure‐tone average.

^a^ Mean (SD); *n* (%).

^b^ Wilcoxon rank sum test; independent samples *t* test; Pearson's *χ* ^2^ test; Fisher's exact test

**Figure 1:**
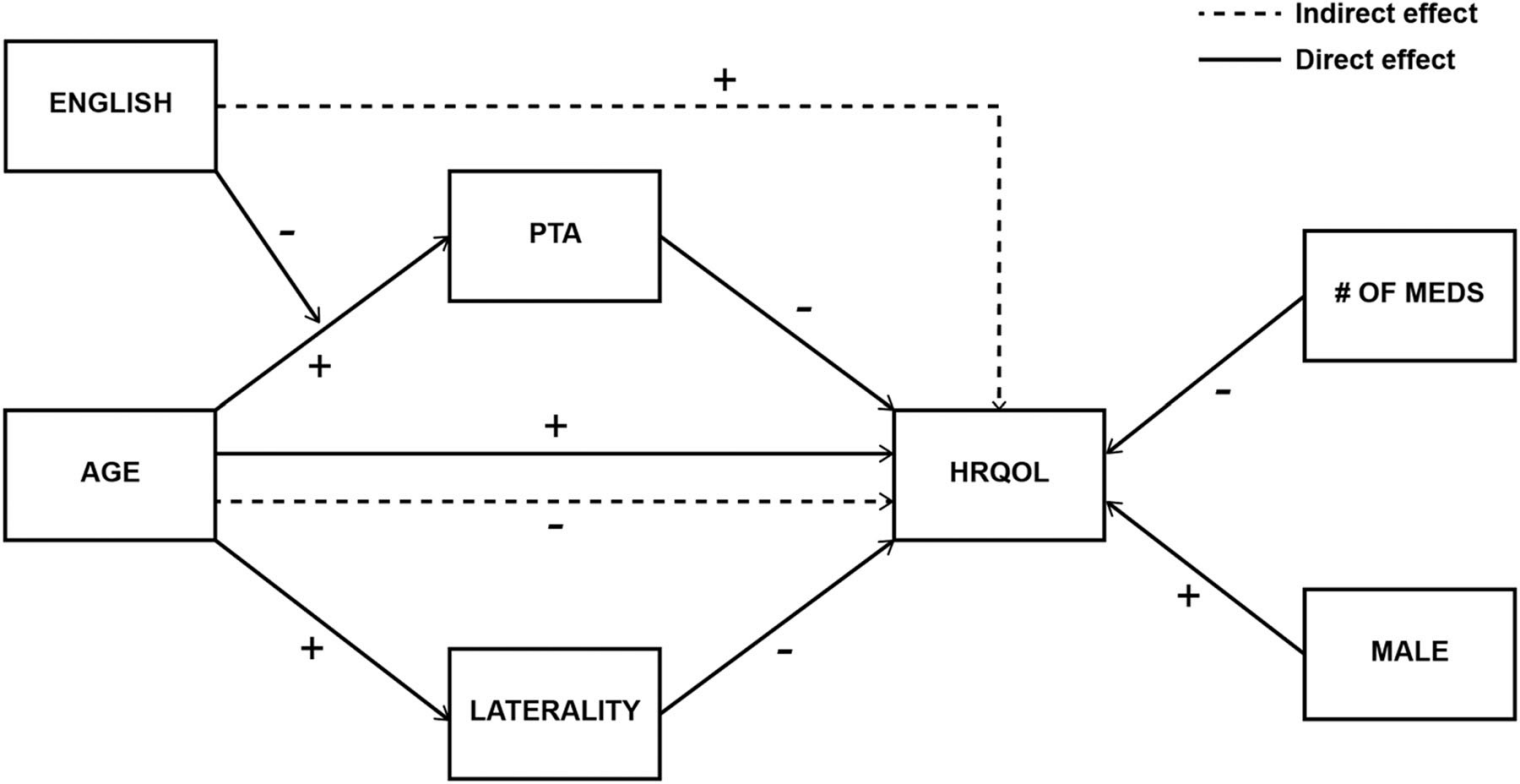
Path model of the direct and indirect effects of personal and clinical factors on health‐related quality of life (HRQoL). PTA, pure‐tone average.

The path model provided an excellent fit ($\chi^{2}$ = 155.65, *p* < .001; CFI = 0.957; RMSEA = 0.055; SRMR = 0.013), explaining 36.5% of the variance in HRQoL. The total, direct and indirect effects of PTA, hearing loss laterality, age, and primary language on HRQoL are provided in Table 2 and Figure 1.

**Table 2:** Parameter Estimates Derived From the Path Analysis

Path	Standardized effects (***β***)		
	Direct effect	Indirect effect	Total
PTA **→** HRQoL	−0.121*	–	−0.121*
Laterality **→** HRQoL	−0.165*	–	−0.165*
Number of medications **→** HRQoL	−0.222**	–	−0.222**
Age **→** HRQoL	0.154*	−0.147*	0.007*
Male **→** HRQoL	0.117*	–	0.117*
English speaking **→** HRQoL	–	0.027*	0.027*
Age × English speaking **→** PTA	−0.226***	–	−0.226***
Age **→** PTA	0.492***	–	
Age **→** LTR	0.530***	–	0.530***

Age × English speaking, the interaction between age and English.

Abbreviations: HRQoL, health‐related quality of life; LTR, hearing loss laterality; PTA, pure‐tone average.

* *p* < .05

** *p* <. 01

*** *p* < .001.

**Figure 2:**
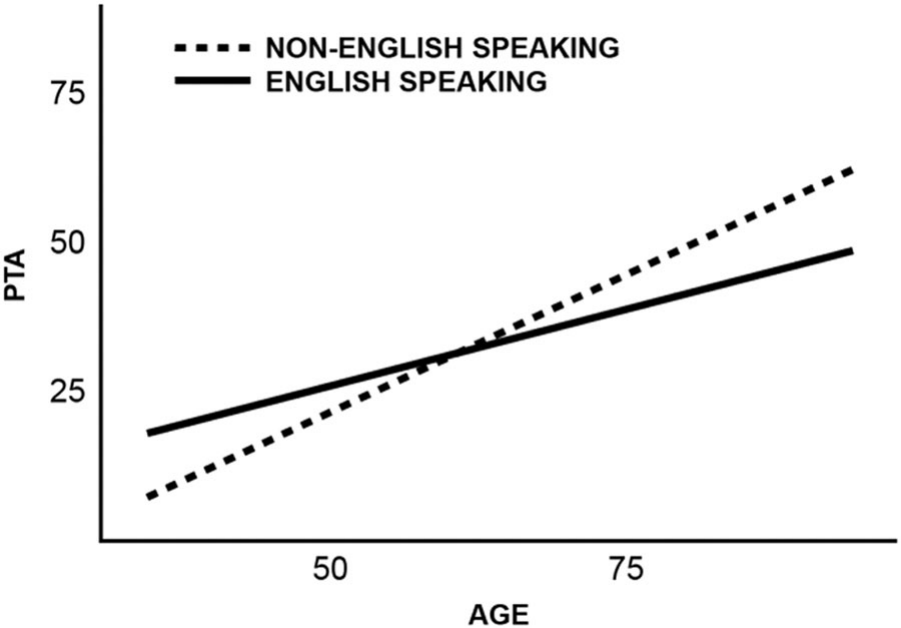
Moderating effect of primary language on the relationship between age and pure‐tone average (PTA).

Both PTA (β = −.18; *p* < .01) and laterality (β = −.26; *p* < .001) had significant direct effects on HRQoL in otology patients, specifically worse hearing and BHL were both associated with lower HRQoL. In our sample, older patients generally reported higher HRQoL (β = .18; *p* < .05). However, aging indirectly affected the relationships between PTA and hearing loss laterality on HRQoL. Namely, older patients with worse hearing had more rapidly declining HRQoL (β = −0.09; *p* < .05). Similarly, older patients with BHL had more rapidly declining HRQoL than if they had UHL (β = −.15; *p* < .01. Number of medications was the only other clinical risk factor that contributed directly to HRQoL as patients who used more medications were more likely to report worse HRQoL (β = −.21; *p* < .01). The number of otologic symptoms and duration of the symptoms did not have significant effects on HRQoL. Male gender was directly positively associated with HRQoL (β = .12; *p* < .05). Patients who spoke English as their primary language reported having significantly worse hearing (β = −.23; *p* < .001), but not on laterality. The correlation of aging with increasing PTA was significantly greater in patients who did not speak English as a primary language compared to patients who spoke English as a primary language (β = −.61; *p* < .05) (Figure 2).

## Discussion

This is the first study to explore the relationship between age, hearing loss, primary language, and HRQoL among otolaryngology patients with otology symptoms. Our findings provide empirical evidence for the hypothesized relationships between age, hearing loss, primary language, and HRQoL. More specifically, this study confirmed that the effect of age on HRQoL is mediated by hearing loss and that primary language is associated with hearing loss and HRQoL.

Previous studies have reported that age is one of the strongest predictors of hearing loss prevalence and severity.^2^, ^24^ It is also well‐documented that hearing loss negatively affects HRQoL.^5^, ^6^, ^7^, ^8^ but the effect of hearing loss on the association between age and HRQoL has yet to be explored. In our study, the direct and indirect effects of age on HRQoL worked in opposing directions. Older age was directly and positively associated with HRQoL. However, the direction of this association was reversed when combined with hearing loss. Older patients exhibited more severe levels of hearing loss, which was negatively associated with HRQoL. These findings highlight the extent of the disabling effect hearing loss imposes upon aging individuals, thus reinforcing the importance of hearing screenings in older adults to improve HRQoL. Increased awareness and earlier diagnosis of hearing loss would enable earlier interventions and treatment plans focused on attaining less isolation, more independence, and overall less stress, which can ultimately lead to a better quality of life.^25^, ^26^, ^27^

We found that primary language moderated the relationship between age and hearing loss. Specifically, patients who did not speak English as a primary language exhibited significantly worse hearing and therefore worse HRQoL than English‐speaking patients with hearing loss. These results are consistent with a growing body of evidence that links language barriers to worse health outcomes.^28^, ^29^, ^30^, ^31^ One study found that language barriers are associated with unequal access to care and poorer health outcomes.^28^ Even when care is available, health care providers view language barriers as a source of workplace stress that impedes their ability to provide high‐quality care.^32^ Thus, this can result in a greater risk for readmission among patients who do not speak English, possibly due to the difficulty health care providers face in comprehending the needs of the patients as well as the inability of patients to understand treatment plans.^33^ Cultural perceptions of hearing loss also might affect the way patients who speak different languages approach treatment. For example, in China, hearing loss is viewed as a normal mechanism of aging and those who experience hearing loss expect their children to adapt to their disabilities.^34^ A recent study assessed the impact of the individualism‐collectivism concept on hearing aid adoption, noting that people from cultures with higher levels of individualism (ie., United Kingdom, Sweden, and Spain) are more likely to seek assistance for hearing loss than people from cultures with lower levels of individualism (ie, India, Malaysia, China, South Korea, and the Philippines).^35^ Previous literature has shown higher rates of health adversity as a result of socioeconomic hardships among individuals with lower levels of English proficiency,^36^ which have been linked to higher levels of occupational and environmental noise exposure, thus predisposing these individuals to noise‐induced hearing loss.^37^ Our findings highlight the need to address the hearing loss disparity that adversely affects patients who do not speak English as a primary language. Ultimately, it is important to develop more effective strategies of communication with patients of various backgrounds because improved communication is linked with better health outcomes and higher quality of life.^38^ Improved methods of outreach and individualized health plans need to be implemented to address this disparity in HRQoL among patients who do not speak English as a primary language.

While the relationship between primary language and health outcomes has been well‐studied, previous research has done little to investigate how UHL affects health outcomes. In 2018, Golub et al concluded that about 7.2% of adults in the United States suffer from UHL,^39^ yet the topic remains understudied. One study in the United States suggested that UHL might adversely affect HRQoL among children but did not find any significant associations.^40^ Our study found that the laterality of hearing loss imposed another mediating effect between age and HRQoL. As patients aged, they were more likely to exhibit BHL than UHL, and patients with BHL had significantly worse HRQoL than patients with UHL. Patients with UHL can still rely on 1 ear for normal hearing while BHL adversely affects overall hearing, which likely decreases HRQoL to a higher degree than those with UHL. However, further research is necessary to explore the impact of UHL versus BHL loss on patients, as our study does not have the depth of data to assess the difference in the effect of UHL and BHL on HRQoL and we did not collect an extensive medical survey of the study patients' other diseases or other environmental risk factors that could also impact UHL or BHL status. Future studies may extend our findings by specifically testing for UHL versus BHL in patients with hearing loss and utilizing specific measurement methods to analyze the effect of UHL versus BHL on HRQoL.

In our study, polypharmacy was associated with lower HRQoL, which was consistent with previous studies.^41^, ^42^, ^43^ Patients taking many medications are more likely to suffer from multiple comorbidities,^44^ which likely affects HRQoL. Polypharmacy also increases the risk of adverse medication interactions and medication noncompliance.^45^ In addition, patients taking multiple medications tend to believe that they are overmedicating.^45^ Our study confirmed that among otolaryngology patients with otology symptoms, HRQoL was inversely associated with the number of home medications. Thus, there are likely significant benefits with careful reconciliation and eliminating unnecessary medications for patients.

Gender was another risk factor associated with HRQoL in our analysis. When controlled for age, primary language, and hearing loss severity and laterality, we found that females had worse HRQoL than males, which is consistent with previous literature.^46^, ^47^, ^48^, ^49^, ^50^ This gender difference in HRQoL has been attributed to many factors, such as income and education levels.^50^ Future studies may consider or incorporate other socioeconomic factors and examine whether the association between gender and HRQoL may differ according to those factors.

The study has several limitations that may prevent generalization beyond our study sample. First, the cross‐sectional nature of the study design limits the determination of causal relationships among study variables. Future research that is longitudinal with attention to understanding the temporal relationships among those variables is indicated. Second, primary language was used as a predicting variable in this study rather than English proficiency, which is a more specific measure of one's ability to understand English. While primary language was self‐reported and determined by which language was utilized during the patient‐physician interaction, some patients who might not be able to interpret more complex medical language may have indicated English as a primary language, thus allowing for errors in interpretation. For patients who did not speak English, a certified translator was used to communicate the instructions for the audiogram prior to the beginning of the test. Consequently, these tests may have been intrinsically biased against patients who did not speak English as a primary language. This study did not document which patients used certified translators during their interaction with the audiologist or physician. Third, our findings pertaining to UHL and BHL were unable to adjust for possible confounding factors such as noise exposure. Lastly, the SF‐12 was administered in English only. For patients who did not speak English as their primary language, response accuracy may be affected. While previous studies have used the SF‐12 to determine HRQoL in patients with hearing loss, the tool's ability to determine the specific effect of hearing loss on HRQoL has yet to be determined.

## Conclusion

Among patients presenting with otology symptoms, not speaking English as a primary language had a significant interaction with older age, creating a synergistic effect for simultaneously worsening hearing function, which was a significant risk factor for lower subsequent HRQoL. Further research is necessary to evaluate interventions that can adequately address this health disparity in HRQoL among patients who do not speak English as a primary language.

## Author Contributions

**Harrison J. Ma**, study conception, design, and material preparation, data collection, data analysis and interpretation of data, drafting of the manuscript, revision of manuscript; **Francis Reyes Orozco**, study conception, design, and material preparation, data collection, revision of manuscript; **Christine K. Raj**, study conception, design, and material preparation, data collection, revision of manuscript; **Kevin Herrera**, study conception, design, and material preparation, data collection, revision of manuscript; **John C. Parsons**, study conception, design, and material preparation, data collection, revision of the manuscript; **Ian Kim**, study conception, design, and material preparation, data analysis and interpretation of data, drafting of manuscript, revision of manuscript; **Kevin Hur**, study conception, design, and material preparation, data analysis and interpretation of data, revision of the manuscript, supervision.

## Disclosures

### Competing interests

None.

### Funding source

None.
